# Depletion of histone methyltransferase KMT9 inhibits lung cancer cell proliferation by inducing non-apoptotic cell death

**DOI:** 10.1186/s12935-020-1141-2

**Published:** 2020-02-17

**Authors:** Hannah Maria Baumert, Eric Metzger, Matthias Fahrner, Julie George, Roman K. Thomas, Oliver Schilling, Roland Schüle

**Affiliations:** 1 Klinik für Urologie und Zentrale Klinische Forschung, Medizinische Fakultät, Albert-Ludwigs-Universität Freiburg, Universitätsklinikum Freiburg, Freiburg, Germany; 2 Institute for Surgical Pathology, Medical Center – University of Freiburg, Faculty of Medicine, University of Freiburg, Freiburg, Germany; 3 Faculty of Biology, Albert-Ludwigs-University Freiburg, Freiburg, Germany; 4 Spemann Graduate School of Biology and Medicine (SGBM), Albert-Ludwigs-University Freiburg, Freiburg, Germany; 5 Department of Translational Genomics, Center of Integrated Oncology Cologne-Bonn, Medical Faculty, University of Cologne, 50931 Cologne, Germany; 6 Department of Pathology, University Hospital Cologne, 50937 Cologne, Germany; 7 German Cancer Research Center, German Cancer Consortium (DKTK), Heidelberg, Germany; 8 Deutsches Konsortium für Translationale Krebsforschung, Standort Freiburg, Freiburg, Germany; 9 BIOSS Centre of Biological Signalling Studies, Albert-Ludwigs-University Freiburg, Freiburg, Germany; 10 CIBSS Centre for Integrative Biological Signalling Studies, Albert-Ludwigs-University Freiburg, Freiburg, Germany; 11 K-metics GmbH, Freiburg, Germany

**Keywords:** Lung cancer, Non-small cell lung cancer, A549, Epigenetics, Histone methyltransferase, KMT9, Transcriptomics, Proteomics

## Abstract

**Background:**

Lung cancer is the leading cause of cancer related death worldwide. Over the past 15 years no major improvement of survival rates could be accomplished. The recently discovered histone methyltransferase KMT9 that acts as epigenetic regulator of prostate tumor growth has now raised hopes of enabling new cancer therapies. In this study, we aimed to identify the function of KMT9 in lung cancer.

**Methods:**

We unraveled the KMT9 transcriptome and proteome in A549 lung adenocarcinoma cells using RNA-Seq and mass spectrometry and linked them with functional cell culture, real-time proliferation and flow cytometry assays.

**Results:**

We show that KMT9α and -β subunits of KMT9 are expressed in lung cancer tissue and cell lines. Importantly, high levels of KMT9α correlate with poor patient survival. We identified 460 genes that are deregulated at the RNA and protein level upon knock-down of KMT9α in A549 cells. These genes cluster with proliferation, cell cycle and cell death gene sets as well as with subcellular organelles in gene ontology analysis. Knock-down of KMT9α inhibits lung cancer cell proliferation and induces non-apoptotic cell death in A549 cells.

**Conclusions:**

The novel histone methyltransferase KMT9 is crucial for proliferation and survival of lung cancer cells harboring various mutations. Small molecule inhibitors targeting KMT9 therefore should be further examined as potential milestones in modern epigenetic lung cancer therapy.

## Background

Lung cancer is the leading cause of cancer related deaths worldwide with 1.8 million deaths predicted for 2018 [1]. Treatment and prognosis widely vary between patients with aggressive small cell lung cancer and slower progressing non-small cell lung cancer (NSCLC). Non-small cell lung cancer represents 85% of all lung cancers, with the most prominent subtypes being adenocarcinoma and squamous cell carcinoma. Yet, the 5-year survival rate for patients with lung cancer is only 10–20%. New treatment strategies based on deeper understanding of the mechanisms driving a lung cell into malignancy are highly needed.

The discovery of global DNA hypo-methylation in human tumors initially broad epigenetics into the focus of cancer research and today epigenetic alterations are known to contribute to cancer development alongside to genetic mutations [2, 3]. The concept of epigenetics was defined as inheritable changes in gene expression that are not due to any alteration in the DNA sequence and relies on DNA methylation and post-translational histone modifications. The modifications can then recruit effector molecules and chromatin modifiers to alter gene expression patterns [4, 5]. The most abundant histone modifications are acetylation, phosphorylation, ubiquitination and methylation by specific chromatin-modifying enzymes. Writing of methyl marks on lysine residues of histone proteins is performed by histone lysine methyl transferases, of which nine were described to be associated with lung cancer [6]. Until recently, all known histone lysine methyl transferases belonged to the su(var)3-9, enhancer-of-zeste and trithorax (SET) domain family with the only exception being DOT1L that belongs to the seven-β-strand family [7, 8]. Recently, a second histone lysine methyl transferase of the seven-β-strand family named lysine methyl transferase 9 (KMT9) was described [9]. KMT9 is an heterodimeric enzyme formed by the assembly of KMT9α (also known as C21ORF127, HEMK2 [10, 11], MTQ2, N6AMT1 [12], PRMC or PRED28 [13] and KMT9β (also known as TRMT112) [9]. KMT9 mono methylates lysine 12 of histone 4 (H4K12me1) in vitro and in vivo thereby controlling proliferation of prostate tumor cells. In prostate tumor cells, KMT9 was found enriched at promoters of numerous cell cycle regulators and it has been shown that KMT9 controls cell cycle progression. KMT9 depletion severely affects proliferation of androgen receptor-dependent as well as castration- and enzalutamide-resistant prostate cancer cells and xenograft tumors [9]. Yet, the function of KMT9 in lung cancer cells has remained elusive. In the present study, we aimed to characterize the role of the novel histone methyltransferase KMT9 in lung cancer.

## Methods

### Cell culture

A549 and PC-9 cells were cultured in DMEM. NCI-H2087 and NCI-H82 cells were cultured in RPMI 1640. GLC-2 cells were cultured in RPMI 1640 supplemented with 1 mM sodium pyruvate and 50 µM 2-mercaptoethanol. IMR-90 and CRL-7000 cells were cultured in EMEM. All media were supplemented with 10% foetal calf serum, penicillin/streptomycin, plasmocin and glutamine. Cells were cultured in plasmocin containing medium at all times and are mycoplasma-free. IMR-90 cells were obtained from ATCC. The other cell lines were kind gifts from research groups as follows: NCI-H82 from M. Burger, Freiburg; GLC-2 from R. Büttner, Bonn; A549 from M. Witzenrath, Berlin; PC-9 from L.C. Heukamp, Köln; NCI-H2087 from S. Wöhrle, Boehringer-Ingelheim. Certificates of cell line authentication are provided as Additional files 1, 2, 3, 4, 5, 6, 7.

### Transfection with siRNA

Cells were transfected with the indicated siRNAs in presence of DharmaFECT 1 (A549; Dharmacon) or RNAiMAX (GLC-2, PC-9, NCI-H2087; Life Technologies) according to the manufacturer`s instruction with a final siRNA concentration of 65 nM (DharmaFECT 1) or 50 nM (RNAiMAX). The sequences of the siRNAs (Stealth RNAi™ siRNAs; Life Technologies) used in the experiments are as following: siControl: 5′-GAAAGUCCUAGAUCCACACGCAAAU-3′; siKMT9α#1: 5′-ACGCUGUAACAAAGUUCACAUUCAA-3′; siKMT9α#2: 5′-CACGCUGUAACAAAGUUCACAUUCA-3′.

### Western blot analysis

Experiments were performed as previously described [14]. If not declared otherwise, 3 days before harvesting, cells were transfected with siRNA as indicated. The following antibodies were used: anti-KMT9α (#27630, lot 20062017, Schüle Lab), anti-KMT9β (#28358, lot 27022018, Schüle Lab), anti-H4K12me1 (#27429, lot 27062017, Schüle Lab); anti-H4 (ab31830, lot GR3204774-2, abcam); anti-Tubulin (alpha tubulin, #T6074, lot 03714804 V, Sigma), anti-LMNA (sc-20680, lot F2607, Santa Cruz); anti-GAPDH (MAB574, lot 3273148, R&D systems); anti-SMARCA2 (NB100-55308; lot A1; Novus biologicals); anti-TIMP2 (CST#5738, lot 3, Cell signaling); anti-SOD2 (CST#13194, lot 1, Cell signaling); anti-YES1 (#PA5-80243, lot VA2919193, Invitrogen). Proteins from patient tissues were extracted using the Minilys homogenizer (Bertin instruments) and RIPA buffer (1 mM EDTA, 50 mM Tris–HCL pH7.5, SDS 0.1%, NaCl 150 mM, NP-40 1%, Na deoxycholate 1%, protease inhibitor cocktail EDTA-free). Samples were cycled for 15 s at top speed.

### Cellular fractionation

For cellular fractionation, a modified REAP protocol [15] was used. Briefly, cell pellets obtained by scraping were washed in PBS and suspended in 0.1% NP-40/PBS, spun down for 10 s, and the supernatant was taken as cytosolic fraction. Pellets of the remaining nuclear fractions were washed with 0.1% NP-40/PBS, suspended in protein lysate buffer (50 mM Tris–HCl (pH8), 170 mM NaCl, 0.1% NP-40, 0.2 mM DTT, 20% glycerol, complete protease inhibitor EDTA-free (Roche), phosphatase inhibitor cocktail (Sigma)), kept on ice for 10 min and sonicated 90 s. Protein concentrations were determined using Nanodrop ND-1000 (Thermo Fisher). 30 µg cytosolic and 150 µg nuclear extract were mixed with SDS-running buffer and denaturized 10 min at 99 °C. The samples were then loaded onto a 15% acrylamide gel for electrophoresis and analyzed by western blotting. Tubulin and Lamin A were used as cytosolic and nuclear marker proteins respectively.

### Histone extraction

Cells were harvested by scraping, pelleted and washed once in PBS. Pellets were suspended in 200 µl Triton extraction buffer (0.5% Triton-x-100, 0.02% NaN_3_, complete protease inhibitor tablet EDTA-free (Roche), PBS) and kept on ice for 10 min while gently vortexing. Then, samples were centrifuged at 4 °C with 400*g* for 10 min, the supernatants were removed, and the pellets were suspended in 100 µl Triton extraction buffer and centrifuged as before. The pellets were suspended in 50 µl 0.2 N HCl and kept at 4 °C overnight while gently agitating. The samples were then centrifuged as before to extract the histones in the supernatant. Bradford assay (BioRad) was used to determine the concentration of the extracts. 2 µg of histones were mixed with SDS-running buffer and denaturized 10 min at 99 °C. The samples were then loaded onto a 18% acrylamide gel and analyzed by western blotting using 4% BSA in 0.5% PBST for blocking and antibody dilutions.

### Cell proliferation assays

Cell proliferation was determined using the xCELLigence RTCA system (Roche) or by counting with Trypan Blue staining using LUNA™ Automated Cell Counter (Logos Biosystems). Real-time recording of cell proliferation with xCELLigence RTCA system was started 24 h after transfection with the indicated siRNAs. For A549 cells 2500 cells/well were seeded in 16 well E-plates (Roche). For GLC-2 15000 cells/well were used. For PC-9 and NCI-H2087 20000 cells/well were used. Cell indices were automatically recorded every 15 min. For Trypan Blue proliferation assay 5 × 10^4^ cells/well (siControl) or 1 × 10^5^ cells/well (siKMT9α#1) were seeded onto 6-well plates 24 h after treatment with siRNA. After 48 h, 72 h and 96 h, supernatant, PBS from washing step and adherent cells harvested by trypsinization were counted and analyzed for viability using Trypan Blue staining and LUNA™ Automated Cell Counter (Logos Biosystems). Population doubling time was calculated from 0 to 72 h (logarithmic growth) using Roth V. 2006 Doubling Time Computing [16].

### Flow cytometry for detection of apoptosis, cell cycle and granularity

For apoptosis and cell cycle analysis, cells were trypsinized 24 h after treatment with siRNA and plated onto 6-well plates. One fraction was analyzed directly (day 1) and two more on day 2 and day 3. For apoptosis analysis cells were after trypsinization, washed in flow cytometry buffer (2% FCS, 2 mM EDTA, PBS) and stained with Annexin V-FITC in binding buffer for 30 min at room temperature. 7-AAD was added 10 min prior to analysis. Reagents were used according to the manufacturer’s protocol (Biolegend, Apoptosis Detection Kit). Cells double positive for Annexin V and PI were considered apoptotic. Cell cycle phase distribution was measured via DNA staining by propidium iodide (PI). To this end, trypsinized cells were washed in PBS, resuspended in 100 µl PBS, fixed by adding 1 ml ice-cold 70% ethanol dropwise while vortexing and kept at − 20 °C for at least 2 h prior to two wash steps in PBS (centrifuged with 500*g*, 3 min at room temperature). Staining was performed in 100 µl PBS with 100 μg/ml RNAse A (Sigma) and 50 μg/ml PI (Sigma) for 30 min at room temperature. Subsequently, cells were analyzed by recording at least 10,000 events and gated in PI-Area versus PI-Width channels. Cell cycle phase proportions were calculated by FlowJo 10.4 software. Cell granularity was measured at day 4 after transfection. In side scatter (SSC) versus forward scatter (FSC) contour plots (10% level) of living single cells a gate “high granularity” was drawn just above the main population of cells treated with siControl and transferred to the cells treated with siKMT9α to quantify population shift in SSC. Flow cytometry was done using BD LSR-Fortessa Cell Analyzer and data were analyzed with FlowJo software.

### RNA isolation

Three days after transfection with siRNA as indicated, cells were harvested by scraping, washed in PBS and resuspended in 500 µl TRIzol. Upon addition of 300 µl chloroform samples were vortexed for 30 s and centrifuged 15 min at 4 °C with full speed. Then, the upper phase was transferred into a new tube containing 500 µl isopropanol. The samples were vortexed 10 s, incubated for 1 h at room temperature and centrifuged as before. Upon removal of the supernatants pellets were sequentially washed with 100% ethanol, followed by 75% ethanol in DEPC-treated H_2_O and allowed to air dry (approximately 30 min). Finally, pellets were resuspended in 15 µl DEPC-H_2_O and incubated for 10 min at 55 °C. RNA concentration was measured by Nanodrop ND-1000 (Thermo Fisher).

### RNA sequencing (RNA-Seq)

RNA samples were sequenced by the standard Illumina protocol to create raw sequence files (.fastq files) at the sequencing core facility of the DKFZ, Heidelberg. Reads were aligned to the hg19 build of the human genome using STAR version 2.5 [17]. The aligned reads were counted with the homer software (analyzeRepeats) and differentially expressed genes were identified using EdgeR [18]. *P* value < 1 * 10^−8^ and log2(fold-change) > 0.26 or log2(fold-change) < (− 0.26) were considered statistically significant. Gene set enrichment analysis (GSEA) was performed using the *Broad Institute* software [19–21], false discovery rate < 10^−5^. Data are deposited under GSE131016.

### Quantitative real-time polymerase chain reaction (qRT-PCR)

Quantitative RT-PCR was performed using the Abgene SYBR Green PCR kit (Invitrogen) according to the supplier’s protocol. *POLR2A* was used for normalization. Primers for *KMT9*α, *YES1, SOD2, TIMP2* and *SMARCA2* were as follows: *KMT9*α (5′-ACGTTTCTGCTTTTGGACGC-3′, 5′-TCAGTGCACATGTACAAAGCC-3′); *YES1* (5′-CGCCTGCAGATTCCATTCAG-3′, 5′-GTCACCCCTTATCTCATCCCA-3′); *SOD2* (5′-TTTCAATAAGGAACGGGGACAC-3′, 5′-GTGCTCCCACACATCAATCC-3′); *TIMP2* (5′-AAGCGGTCAGTGAGAAGGAAG-3′, 5′-GGGGCCGTGTAGATAAACTCTAT-3′); *SMARCA2* (5′-GAAGCACCCAAAGCAAGTCA-3′, 5′-TTCTCTTCGGTTTCCTGCCT-3′).

### Proteomic sample preparation

A549 cells transfected with siControl or siKMT9α#1 (n = 4) were harvested 3 days after transfection. To ensure complete removal of cell culture media the cells were washed twice with 1 ml PBS each time followed by centrifugation at 800*g*. Cell pellets were lysed using 300 µl lysis buffer for 10 min at 95 °C and 750 rpm. The lysis buffer contained 0.1% RapiGest SF (Waters, Milford, MA), 0.1 M HEPES pH 8.0 (AppliChem, Darmstadt, Germany) and protease inhibitors: 10 µM trans-epoxysuccinyl-l-leucylamido (4-guanidino)butane (E-64), 10 µM E64d, 10 mM phenylmethanesulfonyl fluoride (PMSF), 5 mM ethylenediaminetetraacetic acid (EDTA) [22]. Samples were additionally sonicated using 20 cycles (30 s ON/OFF) at 4 °C in a Bioruptor device (Diagenode, Liège, Belgium). Afterwards, the protein concentration was determined using the bicinchoninic acid (BCA) assay (Thermo Scientific) and 100 µg of protein from each sample was transferred to a fresh tube. Protein reduction was performed using 5 mM dithiothreitol (DTT) (AppliChem, Darmstadt, Germany, 15 min at 37 °C, 750 rpm agitation). Free thiols were alkylated by 15 mM 2-iodoacetamide (IAM) and incubated for 15 min at 37 °C in the dark without mixing. Tryptic in-solution digestion was performed by adding sequencing grade trypsin (Worthington, 1:25 enzyme:protein ratio) to each sample and incubating at 50 °C for 2 h at 750 rpm agitation [23] followed by a second digestion step at 37 °C, 18 h at 500 rpm. Samples were then acidified by adding trifluoroacetic acid (TFA) to a final concentration of 2% and incubated for 30 min at 37 °C to degrade the acid-labile surfactant RapiGestSF. All samples were desalted using iST columns according to the manufacturers protocol (PreOmics, Martinsried, Germany) [24]. The peptide concentration was measured using BCA and 25 µg of each sample was transferred to a fresh microreaction tube and vacuum-concentrated until dryness. Samples were resuspended in 50 µl 0.1 M HEPES pH 8.0. For internal quantification control the samples were divided in two groups and different amounts (600 fmol vs. 1000 fmol) of 11 synthetic peptides were spiked-in [25]. Samples including the synthetic peptides were labeled using TMT-10-plex (Thermo Scientific) at room temperature overnight with 550 rpm agitation [26]. All samples were combined and 45 µg of the mixture were fractionated by high pH reversed phase chromatography [XBridge C18 column, 150 mm × 1 mm column containing 3.5 µm particles (Waters)]. An increasing linear gradient of acetonitrile from 10 to 45% over 45 min at a flowrate of 42 µl/min was applied using an Agilent 1100 HPLC system. 24 fractions were collected and concatenated into 12 fractions (combining fraction 1 with fraction 13, fraction 2 with fraction 14 and so on). The resulting 12 fractions were vacuum-concentrated until dryness and stored at − 80 °C until LC–MS/MS analysis.

### Liquid chromatography–tandem mass spectrometry

Each fraction was dissolved in 40 µl buffer A containing 0.1% formic acid in water (Honeywell) and sonicated for 5 min in a sonication water bath. Samples were separated using an EASY-nLC™ 1000 UHPLC system (Thermo Scientific) at a flow rate of 200 nl/min. Prior to the separation, the injected sample (6 µl per fraction) was trapped on a PepMAP100 C18 nanoViper trapping column (20 mm × 75 µm, 3 µm particles). The analytical column was an EASY-Spray™ C18 column (250 mm × 75 µm, 2 µm particles heated at 50 °C). For peptide separation, we used a linear gradient of increasing buffer B (0.1% formic acid in acetonitrile, Fluka), ranging from 5 to 25% acetonitrile over the first 60 min and from 25 to 60% acetonitrile in the subsequent 30 min (90 min separating gradient length). Peptides were analyzed using a Q-Exactive Plus mass spectrometer (Thermo Scientific, San Jose, CA) operating in a data dependent acquisition (DDA) mode. Survey scans were performed at 70,000 resolution, an AGC target of 3e6 and a maximum Injection time of 50 ms followed by targeting the top 10 precursor ions for fragmentation scans at 35,000 resolution with 1.2 m/z isolation windows, an NCE of 32 and an dynamic exclusion time of 40 s. For all ms2 scans the intensity threshold was set to 1000, the AGC to 1e5, maximum Injection time of 100 ms and the fixed first mass to 100 m/z. The mass spectrometry proteomics data have been deposited to the ProteomeXchange Consortium [27] via the PRIDE [28] partner repository with the dataset identifier PXD014145.

### Liquid chromatography–tandem mass spectrometry data analysis

Raw data were analyzed using MaxQuant (v 1.6.0.2) and a human proteome database (reviewed sequences, downloaded from uniprot.org on June 6th, 2017, containing 20188 entries and 11 additional sequences for the iRT peptides). In MaxQuant the PIF was set to 0.75 and up to two missed cleavages were allowed using the Trypsin/P enzymatic specificity. Protein N-term acetylation and oxidation of methionine were set as variable modifications whereas carbamidomethylation of cysteine was set as a fixed modification. Unique and razor peptides were used for protein quantification, allowing for protein quantification based on only one peptide. The results were further processed using R (v 3.5.2) and RStudio (v 1.1.456). First all contaminants, reverse hits and spiked-in peptides were removed as well as proteins which were only identified by site. Subsequently, protein intensities were log2 transformed and normalized across all samples using median polish. To identify differentially expressed proteins, we used the limma package (v 3.40.0). Only proteins with an adjusted P-value < 0.05 were considered as being significantly dysregulated. Those proteins were then combined with the genes found significantly dysregulated in the RNA-Seq experiments resulting in a comprehensive dataset containing transcriptome and proteome information.

### Data analysis

TCGA data was accessed and analyzed by using UALCAN [29]. The t test was performed using a PERL script with Comprehensive Perl Archive Network (CPAN) module “Statistics::TTest”. Survival data of GSE26939 data set was obtained and analyzed using PROGgeneV2 program [30, 31]. Briefly, hazard ratio and confidence interval were determined by cox proportional hazards analysis using ‘coxph’ function in R library ‘survival’, the Kaplan–Meier plot was created using ‘surfit’ function in R library ‘survival’. Prism 6 was used for statistical calculations. Data are presented as means ± standard deviation or means + standard deviation. Significance was calculated by two-tailed paired t test.

## Results

**Fig. 1:**
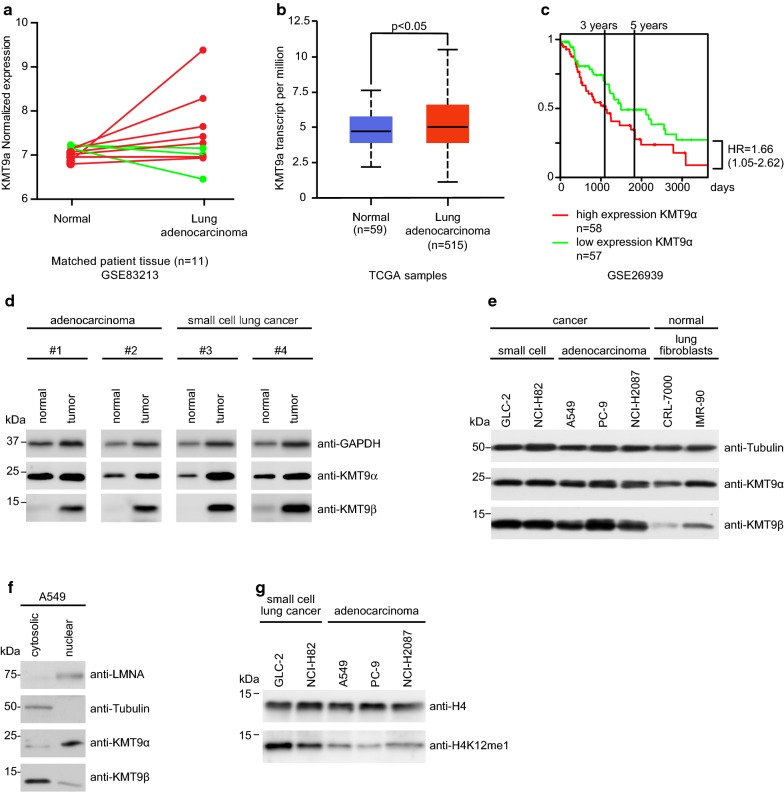
KMT9 is expressed in lung cancer tissue and cell lines. **a** Dynamics of *KMT9α* expression in matched normal and stage 1a lung adenocarcinoma tissue from eleven patients that underwent curative lobectomy. Normal samples were taken at 6 cm distance from macroscopic tumor sites. Data were extracted from (GSE83213). Red lines indicate increased expression of *KMT9α* in tumor (n = 8), green lines indicate decreased expression of *KMT9α* in tumor (n = 3). **b** TCGA data comparing *KMT9α* expression in n = 515 lung adenocarcinoma with non-matched normal lung tissue (n = 59). Data represent interquartile range including minimum, 25th percentile, median, 75th percentile and maximum values. Significance was accessed by t test. **c** Kaplan–Meier survival analysis of patients with adenocarcinoma expressing high (n = 58) and low (n = 57) *KMT9α*. Data were extracted from GSE26939. HR = hazard ratio. **d** Western blots of matched tissue from normal and tumor samples from patients with adenocarcinoma (#1 and #2) or SCLC (#3 and #4). Western blots were performed with the indicated antibodies. **e** Expression levels of KMT9α and KMT9β in human cell lines from SCLC (GLC-2 and NCI-H82), adenocarcinoma (A549, PC-9 and NCI-H2087) and human immortalized normal lung fibroblasts (CRL-7000 and IMR-90) were analyzed by western blot using the indicated antibodies. **f** In A549 cells, KMT9α and KMT9β are present in both nuclear and cytoplasmic compartments. Western blots were performed with the indicated antibodies. **g** Levels of H4K12me1 in SCLC (GLC-2 and NCI-H82) and adenocarcinoma (A549, PC-9 and NCI-H2087) cells were analyzed by western blotting using the indicated antibodies

To address *KMT9* expression in lung cancer we analyzed RNA-Seq data of matched normal and stage 1a lung adenocarcinoma tissue from eleven patients (GSE83213) and found that *KMT9*α expression was increased in seven tumor samples (Fig. 1a). This initial observation prompted us to analyze larger cohorts such as the TCGA cohort presented in Fig. 1b. There, *KMT9*α expression in lung adenocarcinoma is significantly increased compared to normal lung tissue in unpaired samples. Additional file 8a shows *KMT9*α expression for individual tumor stage in the TCGA cohort with a significant increase of *KMT9*α in stage 1 and stage 3. Additional file 8b shows *KMT9*α expression for individual histopathologic subtypes of lung adenocarcinoma. The clinical relevance of KMT9α in lung cancer was revealed by analyzing a cohort of patients with lung adenocarcinoma divided into high expression (n = 58) and low expression (n = 57) of *KMT9α* (GSE26939). As shown in Fig. 1c, patients with low expression of *KMT9α* in their tumor have a higher 5-year and overall survival rate than patients with high expression of *KMT9α* [32]. In Fig. 1d we show that protein levels of KMT9 α and β are increased in lung cancer tissue compared to patient-matched normal lung tissue. Next, we wondered whether KMT9 is present in lung cancer cells. Our western blot analysis in Fig. 1e indicate that both KMT9α and KMT9β are present in small cell lung cancer (GLC-2 and NCI-H82) and NSCLC adenocarcinoma (A549, PC-9 and NCI-H2087) cells with various mutations such as KRAS and CDKN2A mutated A549; CDKN2A, EGFR and TP53 mutated PC-9; and BRAF and TP53 mutated NCI-H2087 cells [33]. Immortalized lung fibroblast cell lines (IMR-90 and CRL-7000) were included as a non-cancer control and reveal equal amounts of KMT9α and a slightly reduced expression of KMT9β. We investigated the cellular distribution of KMT9 in the lung adenocarcinoma cell line A549 cells. Upon cell fractionation, lysates of cytosolic and nuclear fraction were analyzed by western blot. As shown in Fig. 1f, KMT9α and KMT9β were found present in the cytoplasm and the nucleus of A549 cells. Since KMT9 writes the histone mark H4K12me1, we wondered whether the mark is present in various lung cancer cells. Western blot analysis revealed presence of H4K12me1 in the analyzed lung cancer cells (Fig. 1g). Here we show that KMT9 is present in lung cancer tissue and cell lines and that the level of KMT9 in lung adenocarcinoma accounts for patient survival.

**Fig. 2:**
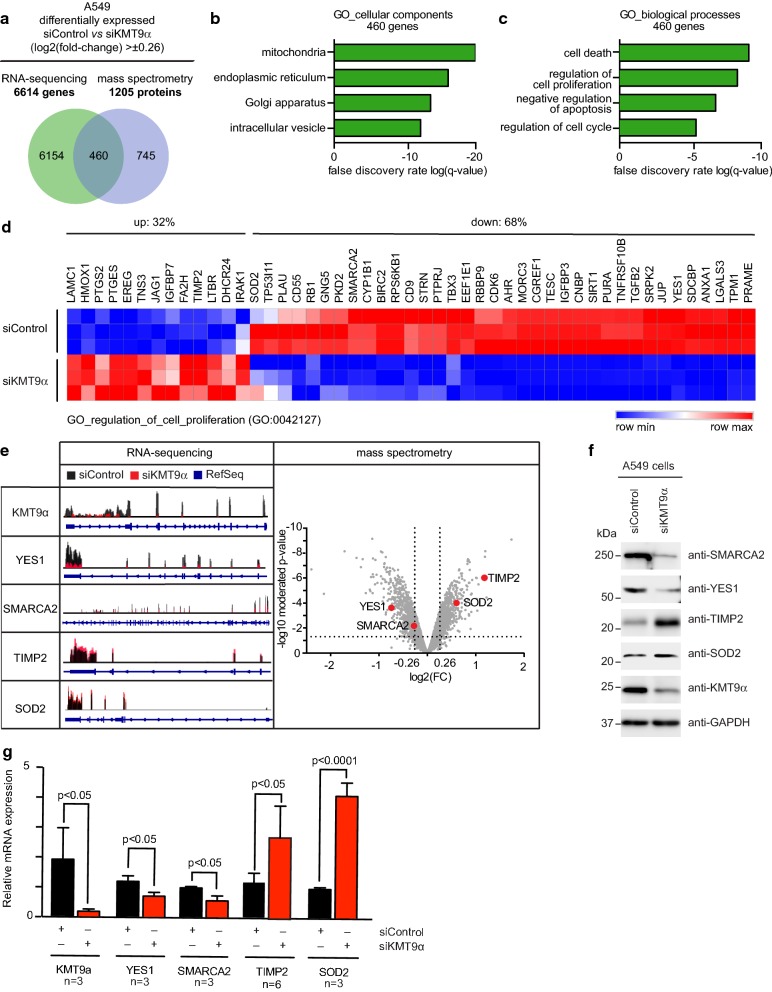
KMT9 controls expression of genes involved in the organization of organelles, cells death and cell proliferation. **a** Venn diagram showing overlap and number of genes/proteins in A549 cells that are differentially expressed upon RNAi mediated knock-down of KMT9α (log2(fold-change) > ± 0.26). In total, 460 targets are concomitantly up- or down-regulated on RNA and protein level upon knock-down of KMT9α. Enriched GO_cellular components **b** and GO_biological processes **c** gene sets obtained for the indicated 460 KMT9α-regulated target genes. **d** Heat map displaying mRNA levels of the 460 KMT9α-regulated genes involved in cell proliferation (GO:0042127) in A549 cells treated with siControl or siKMT9α#1. **e** RNA sequencing reads (left panel) and mass spectrometry volcano plot (right panel) for the indicated genes and proteins are represented exemplarily. **f** Western blot displaying expression of the target proteins indicated in **e** upon knock-down of KMT9α in A549 cells. The indicated antibodies were used. **g** Quantitative real-time PCR analysis of the mRNA expression of the target genes displayed in **e** after knock-down of KMT9α. Data represent means + standard deviation. Significance was accessed by two-tailed t test, n = 3 (TIMP2 n = 6)

Since KMT9 has been shown to regulate gene expression [9] we next performed global transcriptome analyses (RNA-Seq) in A549 cells treated with siControl or siKMT9α#1 and identified 6614 differentially expressed genes upon KMT9α knock-down (Fig. 2a). In parallel, we also performed a global, quantitative proteome analysis (using tandem mass tag multiplexing) in A549 cells treated with siControl or siKMT9α#1. 1205 proteins displayed significant (limma moderated t-statistics) quantitative changes upon KMT9α knock-down (Fig. 2a). Intersection of the transcriptome and the proteome data unraveled 460 targets being up- or down-regulated concomitantly on both mRNA and protein level (Fig. 2a, e). We focused on this concomitantly regulated core set of genes in order to primarily investigate KMT9-dependent expression alterations for which the affected mRNA levels display strong penetration into the proteome level.

To decipher the biological functions of these 460 target genes, we performed gene enrichment analyses. The enrichment analysis for cellular components (Fig. 2b) uncovered genes involved in subcellular organelles such as “mitochondria”, “endoplasmic reticulum”, “Golgi apparatus” or “intracellular vesicles” The enrichment analysis for biological processes (Fig. 2c) revealed genes accounting for “cell death”, “regulation of proliferation”, as well as “regulation of cell cycle” among the top-ranking categories. RNA-Seq counts for the covered genes of the “GO_regulation_of_cell_proliferation” gene set are displayed as a heat map in Fig. 2d. Individual RNA-Seq and mass spectrometry data for important regulatory downstream targets of KMT9 are provided in Fig. 2e. These target genes and proteins were additionally validated by western blot (Fig. 2f) and qRT-PCR (Fig. 2g) analyses. We show that KMT9 controls expression of genes involved in the organization of organelles and regulating cell proliferation.

**Fig. 3:**
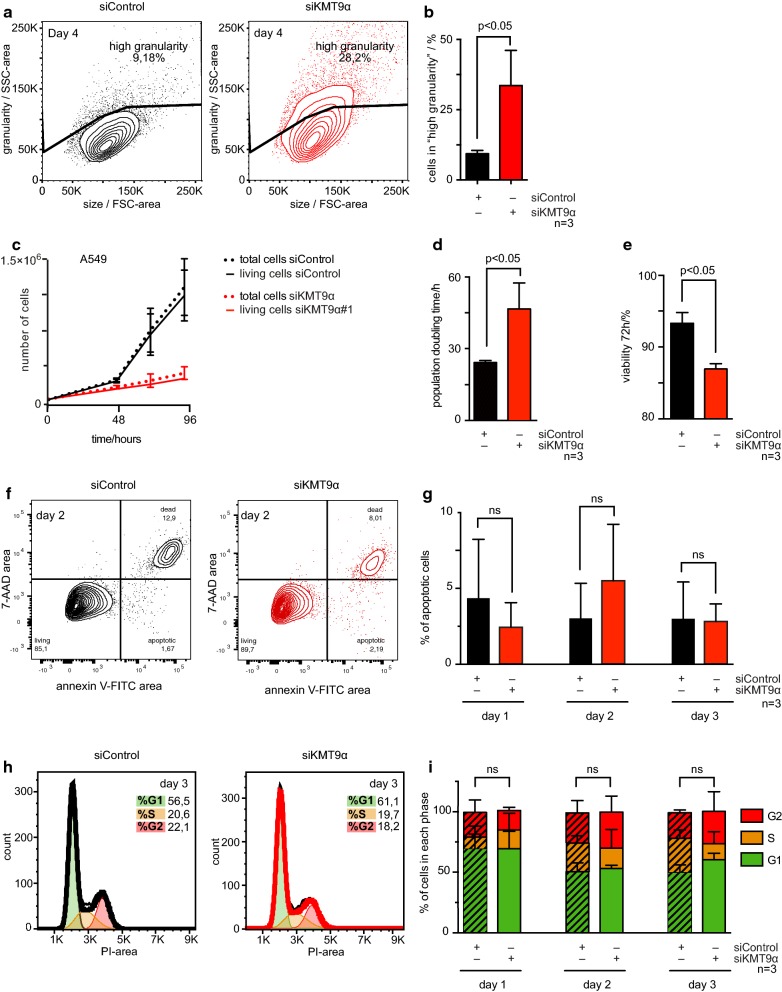
Knock-down of KMT9α inhibits A549 lung cancer cell proliferation and induces non-apoptotic cell death. **a**, **b** The granularity of A549 cells with siControl or siKMT9α#1 was measured by flow cytometry using side scatter (SSC). **a** Figure exemplifying the gating strategy used to assess size of “high granularity” population of A549 cells treated with siControl and siKMT9α#1. **b** Column graph showing the percentage of A549 cells with “high granularity” upon treatment with siControl or siKMT9α#1. Data represent means + standard deviation. Significance was accessed by two-tailed paired t test, n = 3. **c**–**e** Proliferation assay. **c** Representative proliferation curve of A549 cells treated with siControl or siKMT9α#1. Data represent mean ± standard deviation. The experiment was repeated three times independently. **d** Column graph representing the population doubling time (0–72 h). Data represent means + standard deviation. Significance was accessed by two-tailed paired t test, n = 3. **e** Column graph representing the percentage of living cells 72 h after starting the experiment. Data represent means + standard deviation. Significance was accessed by two-tailed paired t test, n = 3. **f**, **g** Apoptosis assay. Apoptotic A549 cells treated with siControl or siKMT9α#1 were identified by flow cytometry using Annexin V and 7-AAD staining. **f** Day 2 of one representative experiment is shown. **g** Bar graphs representing the percentage of A549 apoptotic cells upon treatment with siControl or siKMT9α#1 on days 1–3. Columns represent means + standard deviation. Significance was assessed by two-tailed paired t test, n = 3. **h**, **i** Cell cycle phase distribution was assessed in A549 cells treated with siControl or siKMT9α#1 by propidium iodide (PI) staining and flow cytometry. **h** Day 3 of one representative experiment is shown. **i** Bar graphs representing percentage of cells per cell cycle phase in A549 cells treated with siControl or siKMT9α#1 on days 1–3. Data represent means + standard deviation. Significance was assessed by two-tailed paired t test, n = 3. *SSC* side scatter, *FSC* forward scatter; ns: not significant

To corroborate the transcriptome and the proteome data, we analyzed the biological impact of KMT9 depletion in A549 cells by flow cytometry. As shown in Fig. 3a, b, the percentage of cells showing high granularity increased dramatically upon KMT9α knock-down as would be expected if major changes have occurred in the subcellular organelles. Increase in granularity is associated with physiological processes such as terminal growth arrest and cell death. We therefore measured the population doubling time and the cell viability in A549 cells cultured in the presence of siControl or siKMT9#1. As shown, the population doubling time of A549 cells increased upon knock-down of KMT9α (Fig. 3c, d) and cell viability decreased (Fig. 3e). The experiment shown in Fig. 3f, g indicate that no increase in apoptosis was observed in A549 cells upon siKMT9α mediated knock-down, suggesting that the decrease in cell viability results from non-apoptotic cell death. In addition, no significant alteration of cell cycle phase distribution was detected at day 1, day 2 or day 3 after RNAi transfection (Fig. 3h, i). Here we observed that knock-down of KMT9α in A549 cells leads to inhibition of proliferation and induction of non-apoptotic cell death.

**Fig. 4:**
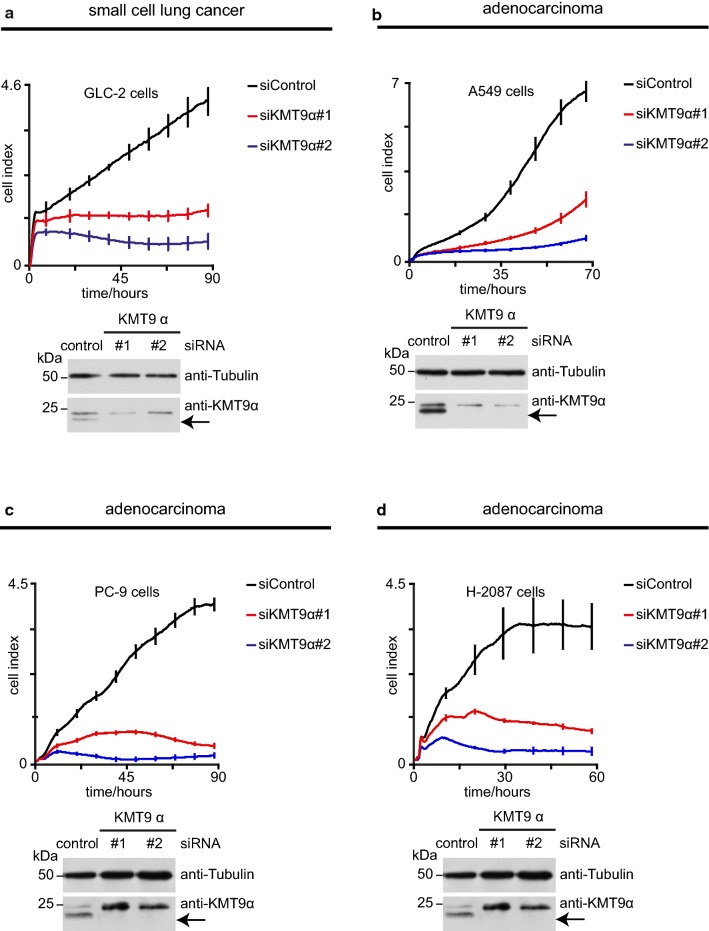
KMT9α controls proliferation of small cell lung cancer and lung adenocarcinoma cell lines. **a**–**d** Real-time proliferation of GLC-2 (**a**), A549 (**b**), PC-9 (**c**) and NCI-H2087 (**d**) cells upon transfection with siControl, siKMT9α#1 or siKMT9α#2. For each cell line one representative experiment is shown (mean ± standard deviation from four technical replicates). Each experiment was performed at least three times independently. Western blot analyses were performed with the indicated antibodies to verify knock-down of KMT9α

Since KMT9 was found to be present in lung cancer cell lines we wondered whether the anti-proliferative effect of siRNA mediated knock-down of KMT9α in A549 lung adenocarcinoma cells could also be observed in other lung cancer cell lines. Therefore, we performed RNAi mediated knock-down of KMT9α in the small cell lung cancer cell line GLC-2 and the adenocarcinoma cell lines A549, PC-9 and NCI-H2087 and monitored real-time proliferation. As shown in Fig. 4a–d, loss of KMT9α severely interferes with proliferation of all tested cell lines. Knock-down efficiency was verified by western blot analyses. These data clearly demonstrate that KMT9 is a global regulator of lung cancer cell proliferation.

## Discussion

We showed that the recently described novel histone methyltransferase KMT9 is expressed in lung cancer cell lines and regulates proliferation and survival of small cell lung cancer and NSCLC lines harboring various mutations.

Recently, Metzger et al. [9] discovered KMT9α to be a novel histone methyltransferase that regulates prostate tumor proliferation. Here, we investigated four lung cancer cell lines and found that upon KMT9α knock-down proliferation of all of them was blocked. In PC-3M prostate cancer cells the anti-proliferative effect was reported to be accompanied by apoptosis and cell cycle arrest and therefore distinct from our observations in A549 cells. This could indicate a role of KMT9 in maintaining the cancer epigenome up-stream of the subtype specific mechanisms by which each cancer cell evades anti-proliferative and cell death signaling. Non-apoptotic cell death, also called regulated necrosis, is of increasing interest in cancer research [34]. Deciphering the exact pathway by which the knock-down of KMT9α leads to non-apoptotic cell death should be investigated in future studies. Recently, Li et al. [35] have correlated N6AMT1 expression with m6dA marks in murine neurons that are associated with activity-induced gene expression. Notably, the experiments by Li et al. were performed in post-mitotic neurons suggesting that the functions of KMT9 include but are not limited to regulating proliferation. Here we report major alterations in gene and protein expression of A549 cells after knock-down of KMT9α indicating the comprehensive role of KMT9 as it would be expected of a bona fide histone methyltransferase. Future research will unravel to what extend KMT9 is a global epigenetic effector in tumor cell proliferation asides from prostate and lung cancer entities.

The development of lung cancer is characterized by continuous genetic and epigenetic alterations. A plethora of driver and passenger mutations has been identified to participate in cancerogenesis of lung cancer. Stage four NSCLC harboring EGFR mutation was the first cancer entity to be treated with a targeted therapy in first line treatment [11, 36, 37]. Today, a growing number of targeted inhibitors are used in molecular treatment of lung cancer depending on the individual mutational status. These therapies are effective but their application is limited to a small patient clientele and in most cases the tumors develop resistance mutations within short notice. Knock-down of the histone methyltransferase KMT9α succeeded to block proliferation of all tested lung cancer cell lines independent of their mutation profile. Targeting chromatin modifying enzymes such as histone methyltransferases represent promising targets for stable therapeutic results in epigenetic therapy and inhibitors of histone methyltransferase EZH2 [38] and histone demethylase LSD1 [39] have already entered clinical trials (NCT03460977, NCT02913443, NCT03337698). Our characterization of the recently discovered histone methyltransferase KMT9 as a prominent regulator of lung cancer cell survival and proliferation paves the way for KMT9 inhibitors to be evaluated as a highly needed additional treatment option in multimodal lung cancer therapy.

## Conclusions

In conclusion, our data demonstrate that the novel histone methyltransferase KMT9 is crucial for proliferation and survival of small cell lung cancer and lung adenocarcinoma cells. Our data link full transcriptome and proteome analyses with functional biological experiments on proliferation and survival thereby identifying 460 genes that are deregulated upon knock-down of KMT9α in A549 cells. These genes cluster with proliferation, cell cycle and cell death gene sets as well as with subcellular organelles in gene ontology analysis. In flow cytometry, we observed major changes in granularity of A549 cells depleted of KMT9α and increased non-apoptotic cell death. Our results are paving the way for small molecules targeting KMT9 to be evaluated as a highly needed new therapeutic approach for lung cancer treatment.

## Supplementary information

**Additional file 1.** Certificate GLC-2. Results and certificate of STR-profiling and cell authentication.

**Additional file 2.** Certificate NCI-H82. Results and certificate of STR-profiling and cell authentication.

**Additional file 3.** Certificate A549. Results and certificate of STR-profiling and cell authentication.

**Additional file 4.** Certificate PC-9. Results and certificate of STR-profiling and cell authentication.

**Additional file 5.** Certificate NCI-H2087. Results and certificate of STR-profiling and cell authentication.

**Additional file 6.** Certificate CRL-7000. Results and certificate of STR-profiling and cell authentication. “HS 1.Lu” is a synonym of CRL-7000.

**Additional file 7.** Certificate IMR-90. Results and certificate of STR-profiling and cell authentication.

**Additional file 8.** KMT9α expression is significantly increased in stage 1 and 3 lung adenocarcinoma from the TCGA cohort. **a** TCGA lung adenocarcinoma samples were divided according to stage and the *KMT9α* expression analyzed. Data represent interquartile range including minimum, 25th percentile, median, 75th percentile and maximum values. Significance was accessed by t test. **b** TCGA lung adenocarcinoma samples were divided according to histopathologic subtypes and the *KMT9α* expression analyzed. Data represent interquartile range including minimum, 25th percentile, median, 75th percentile and maximum values. Significance was accessed by t test. Subgroups with p-value < 0.05 when compared to normal are marked by “*”.

## Data Availability

The transcriptomic dataset generated and analyzed during the current study are available in the GEO repository, GSE131016. The proteomic dataset generated and analyzed during the current study are available in the PRIDE partner repository with the dataset identifier PXD014145.

## Abbreviations

NSCLC: Non-small cell lung cancer
SCLC: Small cell lung cancer
SSC: Side scatter
FSC: Forward scatter
RNA-Seq: RNA sequencing
qRT-PCR: Quantitative real-time polymerase chain reaction
