# Supplementary material for: Depletion of histone methyltransferase KMT9 inhibits lung cancer cell proliferation by inducing non-apoptotic cell death
Source: Cancer Cell Int. 2020 Feb 17;20:52. doi: 10.1186/s12935-020-1141-2 (PMC7027090; doi:10.1186/s12935-020-1141-2)
Supplement: Supplementary file 5 — Additional file 5. Certificate NCI-H2087. Results and certificate of STR-profiling and cell authentication. [file 12935_2020_1141_MOESM5_ESM.pdf]

Eurofins Genomics Europe Applied Genomics GmbH, Anzinger Str. 7 a, D-85560 Ebersberg

Hannah Baumert
Uniklinik Freiburg, Zentrale Klinische Forschung
Breisacher Straße 66
79106 Freiburg
Germany

Certificate
Cell Line Authentication Test
Order ID: 11106693202

Report date: 21.01.2020

Method:
DNA isolation was carried out from cell pellet (cell layer).
Genetic characteristics were determined by PCR-single-locus-technology.
16 independent PCR-systems D8S1179, D21S11, D7S820, CSF1PO, D3S1358, TH01, D13S317, D16S539, D2S1338, AMEL, D5S818, FGA, D19S433, vWA, TPOX and D18S51 were investigated.
(Thermo Fisher, AmpFlSTR® Identifiler® Plus PCR Amplification Kit)
In parallel, positive and negative controls were carried out yielding correct results.

Result:

**Client Sample Name**	7
**Sample Code**	CL00000353
D8S1179	10,12
D21S11	30,32.2
D7S820	8,10
CSF1PO	12,12
D3S1358	15,17
TH01	7,9
D13S317	12,12
D16S539	9,11
D2S1338	20,20
D19S433	14,14
vWA	17,17
TPOX	11,11
D18S51	14,14
AMEL	X,X
D5S818	11,11
FGA	20,20
**Database Name**	NCI-H2087

The table shows the result of the cell line analysis and the comparison with the online database of the DSMZ (http://www.dsmz.de/de/service/services-human-and-animal-cell) and the Cellosaurus database (https://web.expasy.org/cellosaurus). Please note that only the PCR-systems according to ANSI/ATCC standard ASN-0002 were aligned (D5S818, D13S317, D7S820, D16S539, VWA, TH01, TPOX, CSF1PO, AMEL).

This report was created automatically and is therefore valid without a signature.

The laboratory is accredited acc. to **DIN EN ISO/IEC 17025:2005**. The accreditation applies only to the test methods specified in the accreditation certificate. All analyses have been carried out with greatest care and on the basis of state of the art scientific knowledge. The results refer solely to the analysed samples. The duplication and publication also in parts requires a written authorization by this laboratory. Our General Terms and Conditions apply exclusively and are available under eurofinsgenomics.com

DAkkS Deutsche Akkreditierungsstelle D-PL-13372-01-00
