# Supplementary figures and images for: Depletion of histone methyltransferase KMT9 inhibits lung cancer cell proliferation by inducing non-apoptotic cell death
Source: Cancer Cell Int. 2020 Feb 17;20:52. doi: 10.1186/s12935-020-1141-2 (PMC7027090; doi:10.1186/s12935-020-1141-2)

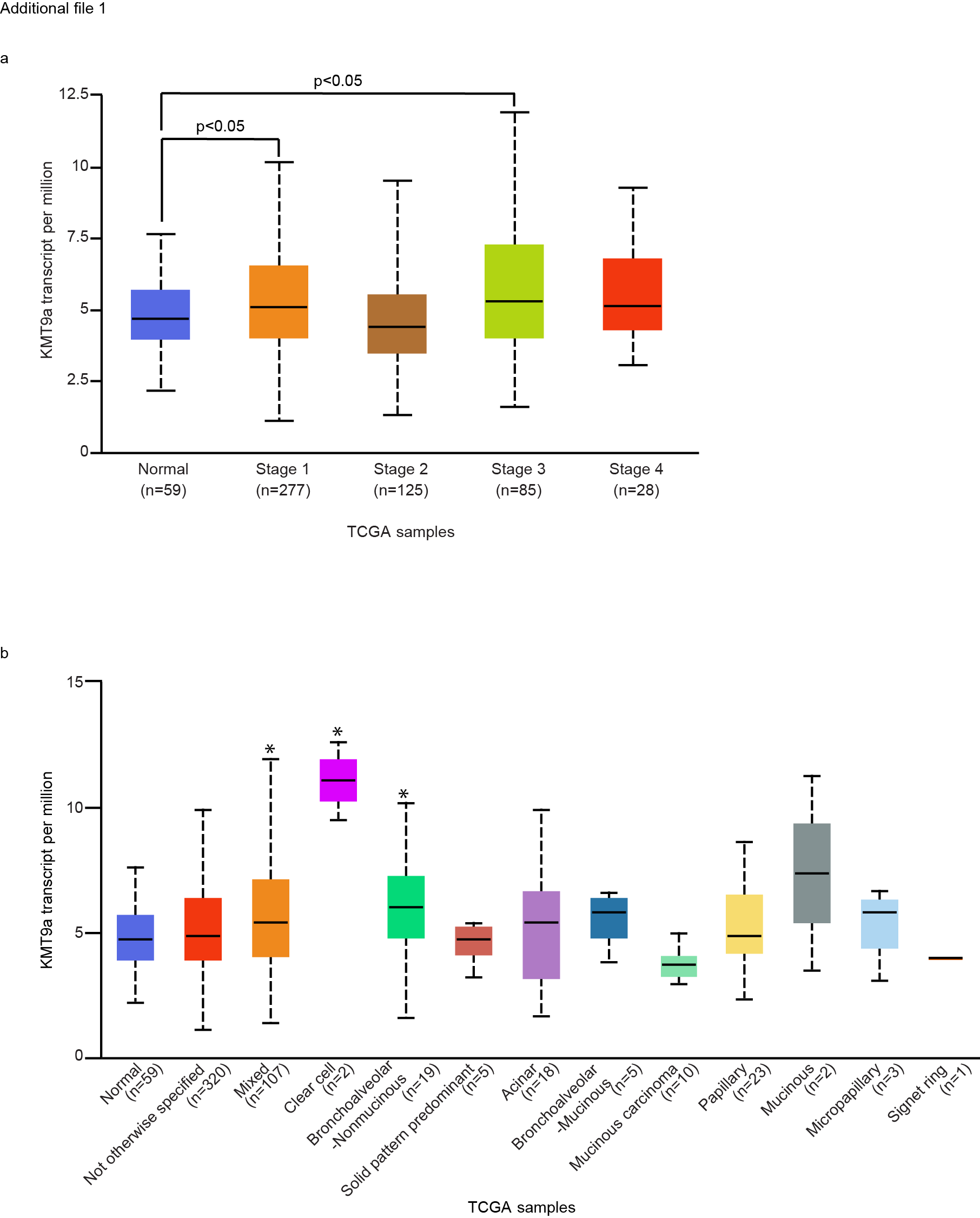

Supplement: Supplementary file 8 — Additional file 8. KMT9α expression is significantly increased in stage 1 and 3 lung adenocarcinoma from the TCGA cohort. a TCGA lung adenocarcinoma samples were divided according to stage and the KMT9α expression analyzed. Data represent interquartile range including minimum, 25th percentile, median, 75th percentile and maximum values. Significance was accessed by t test. b TCGA lung adenocarcinoma samples were divided according to histopathologic subtypes and the KMT9α expression analyzed. Data represent interquartile range including minimum, 25th percentile, median, 75th percentile and maximum values. Significance was accessed by t test. Subgroups with p-value < 0.05 when compared to normal are marked by “*”. [file 12935_2020_1141_MOESM8_ESM.png]
